# Epicardial Adipose Tissue as a Dynamic Modulator of Coronary Artery Disease Phenotypes: Pathophysiology and Therapeutic Implications

**DOI:** 10.1002/clc.70509

**Published:** 2026-10-27

**Authors:** Seyed Amirhossein Dormiani Tabatabaei, Mehrnazsadat Dormiani Tabatabaei

**Affiliations:** ^1^ Isfahan Cardiovascular Research Center, Cardiovascular Research Institute Isfahan University of Medical Sciences Isfahan Iran; ^2^ School of Medicine Isfahan University of Medical Sciences Isfahan Iran

**Keywords:** adipose tissue, atherosclerosis, cardiovascular agents, coronary artery disease, epicardial, inflammation

## Abstract

**Background:**

Epicardial adipose tissue (EAT) represents a localized visceral adipose depot uniquely situated between the myocardium and the visceral layer of the pericardium. Given its proximity to the coronary vasculature and distinct transcriptomic and secretory profile, EAT has been associated with the extent and severity of coronary artery disease (CAD).

**Hypothesis:**

Systemic therapeutic interventions may affect distinct CAD phenotypes through modulation of EAT characteristics.

**Methods:**

This review integrates evidence on EAT pathophysiology and therapeutic modulation in relation to distinct CAD phenotypes. A structured search of PubMed, EMBASE, and the Cochrane Library was conducted using combinations of terms related to EAT, distinct CAD phenotypes, and lifestyle, surgical, and pharmacological interventions. Studies were selected based on their relevance to the review objectives.

**Results:**

EAT has been implicated in a broad spectrum of CAD phenotypes, ranging from microvascular dysfunction to the development of high‐risk atherosclerotic plaque. Advanced imaging modalities enable depot‐specific characterization of EAT as an objectively measurable biomarker. Lifestyle interventions, bariatric surgery, and several pharmacological classes, including glucagon‐like peptide‐1 receptor agonists, sodium–glucose cotransporter‐2 inhibitors, lipid‐lowering agents, and anti‐inflammatory therapies, have been reported to modify EAT.

**Conclusions:**

EAT represents a potential therapeutic target across CAD phenotypes. However, whether therapeutic modification of EAT independently improves cardiovascular outcomes remains uncertain.

## Graphical abstract

Epicardial adipose tissue (EAT) may contribute to the heterogeneous pathophysiology of coronary artery disease (CAD) through interconnected inflammatory, metabolic, and paracrine mechanisms. This review integrates EAT biology across CAD phenotypes and examines therapeutic strategies that may modulate EAT, highlighting current evidence and key gaps in clinical translation.

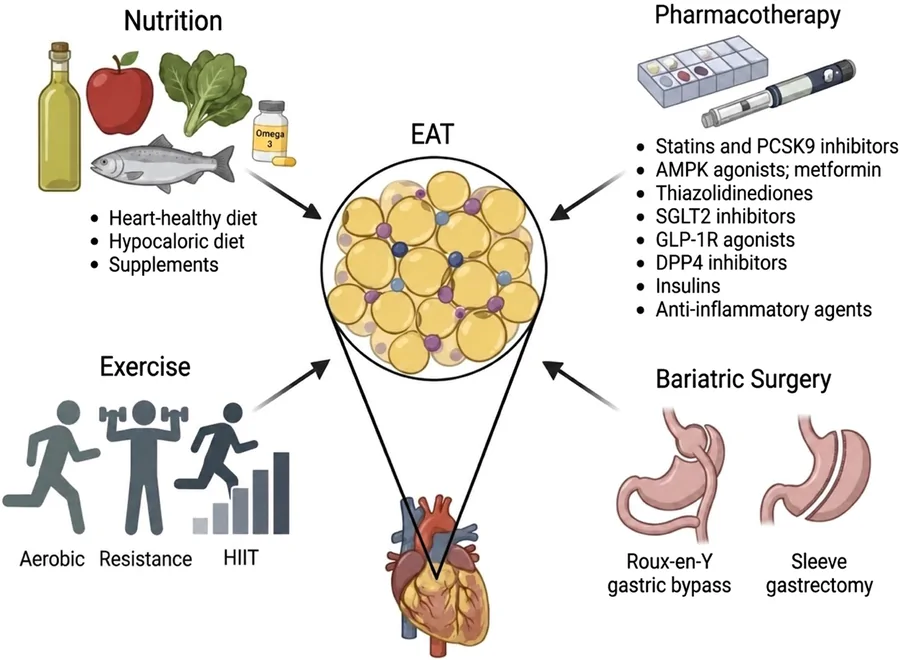

## 1 Introduction

Coronary artery disease (CAD) remains the leading cause of morbidity and mortality worldwide [1]. CAD has long been framed within the paradigm of epicardial coronary artery obstruction, with ≥ 50% angiographic stenosis regarded as the diagnostic gold standard. The understanding of CAD now extends beyond the traditional concept of anatomic epicardial stenosis to a broader range of phenotypes characterized by both structural coronary abnormalities and functional ischemia. This continuum encompasses obstructive ischemic CAD [2]; nonobstructive ischemic syndromes such as ischemia with no obstructive coronary arteries (INOCA) and myocardial infarction with no obstructive coronary arteries (MINOCA) [3]; and plaque‐vulnerability–related CAD [4].

Among emerging mechanisms contributing to CAD heterogeneity, epicardial adipose tissue (EAT) has drawn increasing attention. EAT is a unique adipose depot that envelops the heart and originates from the splanchnic mesoderm [5].

Pericoronary adipose tissue (PCAT), a subset of EAT surrounding the coronary vessels, contains a higher proportion of immature adipocytes and immune cells that interact with adjacent artery segments [6].

Interest in EAT has surged following recent studies indicating that its measurements are independently associated with the extent and severity of CAD, even after adjusting for traditional cardiovascular risk factors. Moreover, EAT has gained prominence as a potential therapeutic target, with evidence suggesting that systemic interventions favorably alter both its quantity and functional activity [5, 7].

However, heterogeneity in CAD presentations limits phenotype‐specific interpretation. With this background, the current narrative review integrates EAT pathobiology with therapeutic modulation of this adipose depot across different CAD phenotypes.

## 2 Pathophysiology

EAT is a highly active depot with distinct secretory and immunometabolic properties. It provides immunological protection and acts as a mechanical cushion that protects coronary arteries against distortion and pulsatile stress. However, under adverse systemic conditions, EAT expands and undergoes pathological remodeling driven by multiple interrelated mechanisms [8].

### 2.1 Heterogeneity of EAT

EAT is not uniformly distributed around the heart. Clinical evidence indicates that the regional distribution of EAT may be as critical as its overall burden. Despite some limitations, the study by Jolfayi et al. examined EAT samples from the right ventricular and atrioventricular regions in patients with and without CAD. EAT from CAD patients exhibited higher resistin expression, lower apelin levels, and a predominance of M1 macrophages [9].

In another study, comparisons of EAT overlying diseased and nondiseased coronary segments showed higher levels of inflammatory mediators in fat adjacent to stenotic segments, pointing to local, segment‐specific adipose activation [10]. In a study of patients with premature CAD, higher EAT volume was associated with greater plaque burden, plaque number, and stenosis severity, while lower EAT density was associated with features of plaque vulnerability, suggesting both EAT expansion and compositional remodeling in aggressive atherosclerosis [11].

In patients with nonobstructive CAD, higher baseline EAT volume was associated with regression of total plaque volume but a shift toward a more vulnerable plaque phenotype during follow‐up, suggesting that EAT may relate to plaque composition beyond overall plaque burden [12].

Likewise, expanded EAT has been associated with lower coronary flow reserve (typically < 2.5), coronary microvascular dysfunction, and nonobstructive ischemic syndromes. Local compressive effects on the coronary microcirculation have been proposed as a potential mechanism [13].

### 2.2 White‐Like Versus Beige/Brown‐Like Adipose Tissue

Epicardial adipocytes exhibit a beige/brown‐like phenotype, rich in mitochondria and relatively increased expression of thermogenesis‐related genes, including UCP1, PRDM16, PGC‑1α, PPARγ, and COX‑IV, compared with other adipose tissues [14].

In its physiological state, beige/brown‐like EAT serves as an immediate energy source, buffers myocardial lipid load, and may exert anti‐inflammatory effects [15].

With advancing age and under comorbid conditions such as central obesity, hypertension, insulin resistance (IR), dyslipidemia, and CAD, EAT undergoes a “whitening” process. As time elapses, it transitions to a pro‐inflammatory, lipid‐storing “white‐like” phenotype that actively secretes atherogenic adipokines (e.g., chemerin, serglycin), loses its thermogenic capacity, and may contribute to vascular inflammation [16].

### 2.3 Adipose–Coronary Crosstalk

The close proximity to the coronary arteries facilitates the transfer of EAT‐derived bioactive factors across the coronary wall via paracrine diffusion or through direct release into the vasa vasorum via vasocrine signaling.

Multiple histopathological reports have described extensive periadventitial inflammation surrounding the spastic coronary artery. Such an inflammatory milieu seems to mediate coronary vasospasm, with the influx of Ca^2+^ through up‐regulated L‐type Ca^2+^ channels as an initial trigger [17]. The diseased EAT shifts its adipokine profile toward decreased levels of cardioprotective adiponectin, adrenomedullin, and omentin‐1 [18]. On the contrary, elevated levels of leptin, visfatin, retinol‐binding protein 4, and lipocalin act via the vasocrine route to inhibit endothelial nitric oxide synthase (eNOS) activity, which has been associated with endothelial dysfunction and increased vulnerability to plaque stress [19]. This adipose–vascular interaction is further amplified by extracellular vesicles (EVs). EVs, particularly exosomes, represent powerful mediators of the adipose–vascular network. Exosomes transport a repertoire of microRNAs, cytokines, bioactive lipids, and regulatory proteins capable of modulating vascular inflammation and immune dynamics in CAD [20]. Notably, adipose‐derived stem cell EVs attenuate myocardial ischemia‐reperfusion (MIR) injury. Under pathological conditions such as EAT overaccumulation or MIR injury, EAT releases pro‐inflammatory EVs that may aggravate local inflammation. Conversely, ischemic myocardium itself increases secretion of pro‐inflammatory EVs, which act on surrounding EAT, potentially favoring whitening of brown/beige adipose cells [21]. In a systematic review of preclinical studies, Shojaei and colleagues highlighted the potential of EV‐based approaches for modulating cardiovascular inflammation [22].

### 2.4 Metabolic Dysregulation

EAT acts as a local source of ectopic lipids, exhibiting a higher rate of fatty‐acid (FA) uptake and release compared to visceral adipose tissue (VAT). Free FA‐related lipotoxicity precipitates mitochondrial dysfunction, impaired oxidative phosphorylation, and elevated generation of reactive oxygen species (ROS) [23]. In systemic metabolic disorders such as obesity and IR, aberrant lipid accumulation fueled by enhanced FA uptake and de novo lipogenesis promotes adipocyte hypertrophy, which contributes to local hypoxia [16]. At the molecular level, the hypoxic state acts as a potent trigger for ROS generation. ROS activates stress‐sensitive kinases alongside the IKK‐β/NF‐κB signaling axis, reinforcing a local inflammatory loop [24]. In patients with CAD, EAT mitochondrial dysfunction correlates with greater severity of coronary atherosclerosis [25]. The increased expression of FA‐binding proteins (e.g., FABP4) and secretory phospholipase A2 in EAT facilitates free FA flux toward the vascular adventitia and intima, potentially promoting atherogenesis. Moreover, inflamed EAT secretes matrix metalloproteinases (MMPs), which degrade the plaque's protective fibrous cap, predisposing the plaque to rupture [16].

### 2.5 Inflammatory Secretory Shift

The EAT immunoregulatory microenvironment consists mainly of dense infiltrates of macrophages, lymphocytes, and mast cells. Notably, EAT expresses NOD‐like receptor protein 3 (NLRP3) inflammasomes. The regional hypoxia initiates an inflammatory secretory shift. Enriched with key inflammatory cytokines and chemokines, this inflamed milieu robustly recruits monocytes via TLR2/4–NF‐κB signaling. In particular, the macrophage balance skews predominantly toward the pro‐inflammatory M1 type. Accordingly, the M1 (CD11c+)/M2 (CD206+) macrophage ratio within EAT is significantly associated with the severity of CAD [26]. Reduced secretion of anti‐inflammatory adipokines coupled with an increase in pro‐inflammatory ones exacerbates macrophage activation and subsequent adaptive immune alterations [9]. These immune alterations are associated with a distinctive histological hallmark, termed crown‐like structures (CLSs) that may exert disproportionate paracrine and vasocrine effects on perivascular segments [27]. Inflammatory adipogenic cells and inflammatory stromal cells were identified as CAD‐enriched populations within EAT, with exploratory imaging data suggesting an association between EAT expansion and cellular inflammatory remodeling [28].

## 3 Therapeutic Implications

Given its rapid metabolism, organ fat specificity, and simple objective quantification possibilities, EAT represents a therapeutic candidate for systemic interventions commonly applied in subjects with diabetes, dyslipidemia, and elevated cardiovascular risk. Importantly, the durability of EAT changes suggests it is a modifiable yet stable target even when underlying metabolic derangements are ameliorated [29]. Multimodality imaging has enabled noninvasive characterization of EAT.

Volumetric assessment of EAT using modalities such as cardiac computed tomography (CCT) and cardiac magnetic resonance imaging (CMRI) is more reproducible and yields higher interobserver agreement than echocardiography‐based thickness measurements [30].

Nevertheless, histological evidence points to the fact that inflammatory activity within EAT is highly heterogeneous and cannot be fully captured by three‐dimensional quantification alone. The CT‐derived EAT attenuation and coronary computed tomography angiography (CCTA)‐derived fat attenuation index (FAI) provide complementary information on EAT characteristics [31].

Longitudinal imaging further helps monitor dynamic changes in EAT in response to therapeutic interventions. However, methodological heterogeneity and the lack of standardized acquisition and analysis protocols remain important barriers to clinical translation.

### 3.1 Nonpharmacological Interventions

Growing evidence supports the role of nonpharmacological interventions in modulating EAT biology and attenuating its pro‐inflammatory and cardiometabolic effects.

#### 3.1.1 Exercise

Exercise training promotes EAT reduction even in the absence of caloric restriction [32]. Randomized controlled trials indicate that both aerobic and resistance training independently diminish EAT mass. These effects often occur in parallel with VAT loss and are not entirely mediated by changes in body weight or body mass index (BMI). Concurrent training regimens may further augment cardiometabolic adaptations; however, clinically meaningful EAT depletion often necessitates a greater magnitude of overall weight loss [33].

Notably, high‐intensity interval training appears to accelerate EAT regression even over short intervention periods, highlighting the depot's sensitivity to acute metabolic stress driven by increased lipolysis, enhanced fatty acid oxidation, and improved mitochondrial function [34].

Ultimately, despite decreased quantity, evidence for exercise‐induced changes in EAT quality remains scarce.

#### 3.1.2 Nutrition

Adherence to heart‐healthy dietary patterns is associated with favorable EAT characteristics. Specifically, the Mediterranean diet appears to be superior to others in reducing EAT, likely owing to its anti‐inflammatory properties and high proportion of healthy fats [35].

Hypocaloric dietary interventions, particularly very low‐calorie diets, consistently correlate with decreased EAT thickness and volume. The pronounced response of EAT to caloric restriction highlights its metabolic flexibility and its close coupling to systemic energy balance [34].

Supplementation with omega‐3 polyunsaturated fatty acids (n‐3 PUFAs) has been shown to increase heme oxygenase‐1 (HO‐1) expression, thereby suppressing NF‐κB signaling. Furthermore, the enhanced production of specialized pro‐resolving mediators, such as Resolvin D1, facilitates the active resolution of pro‐inflammatory profile of EAT [36].

#### 3.1.3 Bariatric Surgery

Bariatric surgery, primarily sleeve gastrectomy and Roux‐en‐Y gastric bypass, has been shown to elicit sustained weight loss coupled with favorable hormonal and metabolic adaptations [37]. It induces substantial diminutions in EAT volume, often surpassing those achieved through lifestyle modifications alone [38].

Typically evident within 3–6 months, the EAT reductions remain relatively modest compared to VAT loss. Nonetheless, it remains a last‐line option for patients with morbid obesity [34].

### 3.2 Pharmacological Interventions

Systemic pharmacological interventions may modulate EAT through multiple metabolic, anti‐inflammatory, and immunomodulatory pathways (Figure 1). A substantial body of evidence suggests that changes in EAT dimensions and attenuation are novel avenues in the pharmacotherapy of diabetes and obesity, with particular focus on cardiovascular risk reduction. However, relatively few radiologic studies have directly evaluated the effects of such agents on EAT (Table 1).

**Figure 1:**
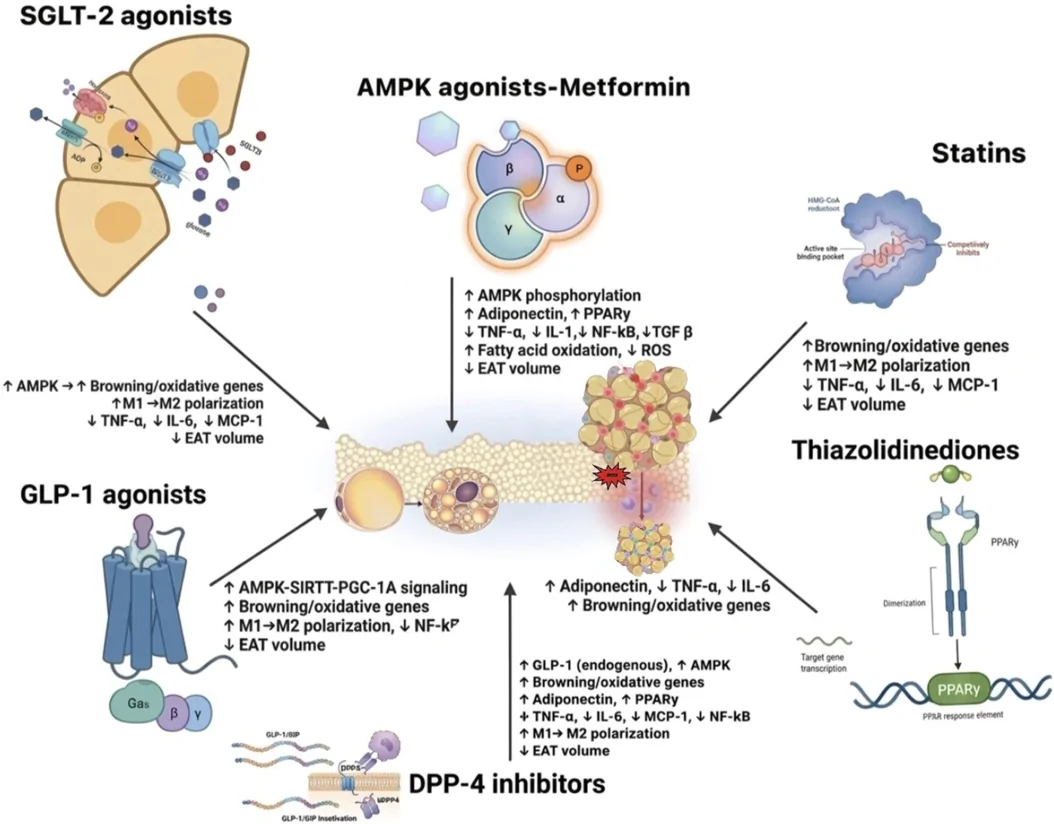
Proposed effects of statins, AMPK agonists–metformin, PPARγ agonists, SGLT2i, GLP‐1R agonists, and DPP‐4 inhibitors on EAT.

**Table 1:** Clinical imaging studies evaluating the effects of pharmacological therapies on EAT.

Class	Intervention	Study design	Study population (*N*)	Adipose depot/endpoint	Modality	Duration (weeks)	Main finding	References
Statins	Atorvastatin (20 mg once daily) Simvastatin (10 mg once daily) + ezetimibe (10 mg once daily)	Retrospective cohort study	Patients with chronic stable angina undergoing PCI (*N* = 145)	EAT thickness	Echocardiography	24–32	Decreased by 0.47 ± 0.65 mm with atorvastatin (11.3%) vs. 0.12 ± 0.52 mm with simvastatin/ezetimibe (3%); *p* = 0.001.	Park et al. [39]
	Atorvastatin (80 mg once daily) Pravastatin (40 mg once daily)	Substudy of a randomized controlled trial	Hyperlipidemic postmenopausal women (*N* = 420)	EAT volume	CCT	52	EAT volume decreased by 3.38% with atorvastatin vs. 0.83% with pravastatin; *p* = 0.025.	Alexopoulos et al. [40]
PCSK9 inhibitors	Evolocumab Alirocumab	Prospective single‐arm observational study (conference abstract)	Patients under treatment of PCSK9 inhibitors (*N* = 24)	EAT thickness	Echocardiography	26	EAT thickness decreased from 6.84 ± 1.97 to 5.41 ± 1.86 mm (−20.39% ± 17.20%); *p* = 0.0001.	Rivas Galvez et al. [41]
Biguanide	Metformin (1000 mg twice daily)	Prospective observational study	Newly diagnosed T2DM (*N* = 40)	EAT thickness	Echocardiography	12	EAT thickness decreased from 5.07 ± 1.33 to 4.76 ± 1.32 mm (6.1%); *p* < 0.001.	Ziyrek et al. [42]
Thiazolidinediones	Pioglitazone (15 then 30 then 45 mg once daily)	Prospective interventional study	Newly diagnosed T2DM (*N* = 24)	EAT area and paracardial adipose tissue area	CMRI	24	EAT area decreased from 15.3 ± 3.9 to 14.0 ± 3.9 cm^2^ (8.5%); *p* = 0.03. Paracardial adipose tissue area decreased from 33.5 ± 13.2 to 30.4 ± 14.1 cm^2^ (9.3%); *p* = 0.09.	Moody et al. [43]
SGLT2 inhibitors	Empagliflozin (10 mg once daily)	Randomized placebo‐controlled trial	Nondiabetic patients with HFrEF (*N* = 84)	EAT volume	CMRI	24	EAT volume decreased from 50.3± 33.2 to 45.2 ± 27.4 mL (10.2%) with empagliflozin vs. 53.1 to 52.3 mL (1.4%) with placebo; between‐group difference *p* < 0.05.	Requena‐Ibáñez et al. [44]
	Empagliflozin (10 mg once daily)	Randomized placebo‐controlled trial	T2DM patients (*N* = 56)	EAT volume	CMRI	12	No significant change in EAT volume.	Gaborit et al. [45]
	Empagliflozin (10 mg once daily)	Post hoc substudy of a randomized placebo‐controlled trial	Patient with HFrEF and T2DM or prediabetes (*N* = 90)	EAT volume	CMRI	36	EAT volume decreased by 2.7 mL (3.1%) with empagliflozin vs. increased by 0.3 mL (1.5%) with placebo; between‐group *p* = 0.045.	Turgut et al. [46]
	Dapagliflozin (10 mg once daily)	Randomized placebo‐controlled trial	T2DM patients without overt CVD (*N* = 56)	EAT thickness	CMRI	48	Average EAT thickness decreased from 6.1 (5.6–7.5) to 5.4 (4.4–6.4) mm (median change, −0.8 mm; 13%) with dapagliflozin vs. 5.8 (4.8–6.9) to 5.6 (4.8–7.3) mm (median change, +0.1 mm) with placebo; *p* < 0.01.	Cha et al. [47]
	Dapagliflozin (10 mg once daily)	Prospective controlled study	T2DM with CAD HbA1C ≥ 6.5% despite treatment with only diet and exercise or oral antidiabetic agents (*N* = 40)	EAT volume	CCT	24	EAT volume decreased by 16.4 ± 8.3 mL with dapagliflozin while increased by +4.7 ± 8.8 mL with conventional treatment (14.3%); *p* = 0.01.	Sato et al. [48]
	Luseogliflozin (2.5–5 mg once daily)	Single‐arm pilot study	T2DM with BMI ≥ 25 kg/m^2^ (*N* = 19)	EAT volume	CMRI	12	EAT volume decreased from 117 to 111 cm^3^ (5.1%); *p* = 0.048.	Bouchi et al. [49]
	Canagliflozin (100 mg once daily)	Single‐arm interventional study	T2DM (*N* = 13)	EAT thickness	Echocardiography	26	EAT thickness decreased from 9.3 ± 2.5 to 7.3 ± 2.0 mm (21.5%); *p* < 0.001.	Yagi et al. [50]
	Ipragliflozin (50 mg once daily)	Single‐arm pilot study	Nonobese T2DM with visceral obesity (*N* = 9)	EAT volume	CMRI	12	Median EAT volume decreased from 102 (79–126) to 89 (66–109) mL (12.7%); *p* = 0.008.	Fukuda et al. [51]
GLP‐1R agonists	Semaglutide (1 mg sc weekly)	Prespecified secondary analysis of a randomized trial	T2DM patients with established CAD (*N* = 90)	EAT volume and density	CCT	48	EAT volume decreased by 10.7 ± 24.6 cm^3^ with semaglutide vs. increased by 6.4 ± 22.8 cm^3^ with placebo; *p* = 0.001. EAT density increased by 4.8 ± 11.1 HU vs. increased by 0.6 ± 10.2 HU, respectively; *p* = 0.036.	Manubolu et al. [52]
	Semaglutide (1 mg sc weekly) Dulaglutide (1.5 mg sc weekly)	Retrospective observational study	T2DM BMI ≥ 27 kg/m^2^ (*N* = 80)	EAT thickness	Echocardiography	12	EAT thickness decreased from 9.5 ± 2.6 to 7.5 ± 2.0 mm (21.1%) with semaglutide and from 9.3 ± 2.2 to 7.7 ± 2.2 mm (17.2%) with dulaglutide; *p* < 0.01 for both.	Iacobellis and Villasante Fricke [53]
	Liraglutide (up‐titrated to 1.8 mg once daily)	Double‐blind randomized placebo‐controlled trial	South Asian patients with T2DM (*N* = 47)	EAT volume	CMRI	26	No significant change in EAT volume.	van Eyk et al. [54]
	Liraglutide (titrated to a maximum dose of 1.8 mg once daily)	Randomized placebo‐controlled trial	T2DM with BMI > 25 kg/m^2^ (*N* = 50)	EAT area	CMRI	26	No significant change in EAT area.	Bizino et al. [55]
	Liraglutide (1.8 mg sc once daily)	Randomized open‐label controlled trial	T2DM patients with BMI ≥ 27 kg/m^2^ HbA1c ≤ 8%, prior treatment exclusively with metformin (*N* = 95)	EAT thickness	Echocardiography	26	EAT thickness decreased from 9.6 ± 2.0 to 6.2 ± 1.5 mm (−36%); *p* < 0.001.	Iacobellis et al. [56]
	Liraglutide (1.2 mg sc daily) vs. Exenatide (5 mcg then 10 mcg sc twice daily)	Prospective comparative interventional study	T2DM patients (*N* = 25)	EAT thickness	Echocardiography	12	EAT thickness decreased from 9.4 ± 1.6 to 8.0 ± 1.9 mm (23.2% ± 13%); *p* = 0.003.	Morano et al. [57]
	Exenatide (5–10 mcg sc twice daily)	Randomized controlled trial	Obese, T2DM subjects uncontrolled on oral antidiabetic drugs (*N* = 44)	EAT volume	CMRI	26	EAT volume decreased by −8.8% ± 2.1% with exenatide vs. −1.2% ± 1.6% with reference treatment; *p* = 0.003.	Dutour et al. [58]
	Liraglutide (1.8 mg sc once daily)	Prospective single‐arm interventional study	T2DM patients with BMI 25.2–37.5 kg/m^2^, HbA1c > 6.5%, WC> 80 cm (F) WC > 90 cm (M) (*N* = 36)	EAT thickness	Echocardiography	12	EAT thickness decreased from 9.14 ± 2.39 to 6.42 ± 1.48 mm (29.8%); *p* < 0.05.	Li et al. [59]
DPP4 inhibitors	Sitagliptin (50 mg twice daily) + metformin (1000 mg twice daily)	Prospective interventional pilot study	T2DM patients inadequately controlled with metformin, HbA1C ≥ 7% (*N* = 26)	EAT thickness	Echocardiography	24	EAT thickness decreased from 9.98 ± 2.63 to 8.10 ± 2.11 mm (reported relative change, 15%); *p* = 0.001.	Lima‐Martínez et al. [60]
Anti‐inflammatory therapies	Biologic (anti TNF, anti‐IL‐12/23, anti‐IL‐17)	Prospective cohort study	Moderate‐to‐severe psoriasis without biologic therapy at baseline (*N* = 134)	Pericoronary FAI	CCTA	52	Median pericoronary FAI decreased from −71.22 HU (IQR, −75.85 to −68.11) to −76.09 HU (IQR, −80.08 to −70.37), corresponding to a Δ −4.87 HU; *p* < 0.001.	Elnabawi et al. [61]
	Monoclonal antibody orticumab	Randomized placebo‐controlled pilot study	Moderate‐to‐severe psoriasis (*N* = 51)	Pericoronary FAI	CCTA	15	Significant reduction in FAI in patients with elevated baseline coronary inflammation on CCTA.	Farina et al. [62]
	Colchicine 0.5 mg/day	Randomized placebo‐controlled trial	Stable CAD patients (*N* = 69)	EAT volume EAT density	CCT/CCTA	52	On CCTEAT volume decreased by 0.1 ± 9.7 mL with colchicine vs. 5.7 ± 11.8 mL increase with placebo, *p* = 0.029 EAT density was unchanged.	Bhandari et al. [63]

Abbreviations: CCT, cardiac computed tomography; CCTA, Coronary computed tomography angiography; CMRI, cardiac magnetic resonance; CVD, cardiovascular disease; HFrEF, heart failure reduced ejection fraction; PCI, percutaneous coronary intervention; WC, waist circumference; T2DM, type 2 diabetes mellitus.

#### 3.2.1 Statins and PCSK9 Inhibitors

Beyond their established lipid‐lowering actions, statins exert a constellation of pleiotropic anti‐inflammatory and metabolic properties that extend to EAT.

Clinical data have shown that statin therapy induces a modest, yet statistically significant reduction in EAT thickness and attenuation independent of changes in serum LDL‐cholesterol, CAC score, or EAT volume [64].

Mechanistically, these agents inhibit VCSMC migration and proliferation, suppress macrophage growth, lower MMP secretion, and impair cell adhesion [65].

Such benefits are underpinned by downregulation of inflammatory signaling, mitigation of oxidative stress, and improvement of endothelial nitric oxide bioavailability. These effects are reflected in dampened paracrine and vasocrine inflammatory crosstalk between EAT and the adjacent coronary arterial wall, contributing to plaque stabilization [66].

These modifications suggest a reorganization of immune infiltrates toward a less pathogenic state, evidenced by reduced secretion of TNF‐α, IL‐6, and MCP‐1, alongside corresponding changes in CT radiodensity [67].

As one of the most comprehensively studied statins, high‐intensity atorvastatin (80 mg/day) consistently leads to a decrease in volume and attenuation of EAT [64].

Notably, atorvastatin at 20 mg/day was reported to be superior to a simvastatin/ezetimibe (10/10 mg/day) combination in reducing EAT thickness [39].

Pravastatin (40 mg/day), predominantly serving as a comparator in clinical studies, showed only a marginal reduction in EAT volume compared with intensive statin therapy with atorvastatin in a substudy of the BELLES trial. This limited efficacy may reflect its hydrophilic nature, which is associated with greater hepatoselectivity and lower extrahepatic tissue penetration. Available data suggest that statin therapy intensity, rather than the specific agent, is a principal determinant of EAT regression [40].

In patients with INOCA, statin use was associated with lower EAT density and attenuated the positive association between EAT density and INOCA risk [68].

Currently, no direct experimental or clinical evidence is available regarding the effects of lovastatin, pitavastatin, or fluvastatin on EAT. Nevertheless, the overall impact remains mild compared with newer lipid‐lowering agents such as proprotein convertase subtilisin/kexin type 9 (PCSK9) inhibitors.

PCSK9 is locally expressed in EAT, where it correlates with pro‐inflammatory mediators and may drive a feed‐forward loop between adipose and vascular inflammation through activation of TLR4/NF‐κB signaling and engagement of the NLRP3 inflammasome [69].

Targeting this pathway, monoclonal antibodies such as evolocumab and alirocumab preliminarily demonstrated substantial reductions in EAT thickness of approximately 20% following a 6‐month treatment period, independent of the extent of LDL‐cholesterol reduction [41].

#### 3.2.2 AMPK Agonists–Metformin

As a first‐line therapy for type 2 diabetes mellitus (T2DM), metformin activates AMP‐activated protein kinase (AMPK) to improve glycemic control by inhibiting hepatic gluconeogenesis, enhancing peripheral glucose uptake, and reducing body weight and VAT [70].

Metformin monotherapy has been shown to regress EAT thickness in individuals with obesity, IR, or newly diagnosed diabetes [42, 71].

Mechanistically, this effect is achieved through enhanced FA oxidation, attenuated lipogenesis, and direct anti‐inflammatory pathways, likely mediated by modulation of MCP‐1, IL‐6, CRP, and NF‐κB, alongside the M1‐type macrophage polarization [72].

In a retrospective study of 547 T2DM patients, metformin use was associated with lower PCAT attenuation on CCTA, indicating mitigated coronary inflammation [73].

Notably, a relatively short‐term treatment of just 3 months yields this effect, occasionally accompanied by reductions in BMI [71].

In contrast, the literature presents some contradictory findings; for instance, certain MRI‐based studies have reported no considerable changes in EAT following metformin treatment, particularly in CAD patients without T2DM [74].

#### 3.2.3 Thiazolidinediones

Thiazolidinediones, which are peroxisome proliferator–activated receptor (PPAR) agonists, promote adipocyte differentiation, enhance insulin sensitivity, and exert profound immunometabolic and volume‐reducing effects on EAT [75].

The PPARγ isoform upregulates PRDM16, PGC‐1α, and UCP‐1, thereby inducing the browning shift of adipose tissue and mitochondrial biogenesis. Higher mitochondrial biogenesis enhances fatty acid oxidation and lipid turnover [76].

In patients with CAD and T2DM, pioglitazone therapy was associated with reduced expression of IL‐1β, IL‐1Ra, and IL‐10 in EAT, although the specific effects on macrophage polarization were not directly assessed [77].

In a small study of nondiabetic patients with stable CAD, pioglitazone increased plasma adiponectin levels and improved endothelium‐dependent flow‐mediated vasodilation [78].

While several clinical studies have demonstrated the efficacy of pioglitazone therapy in reducing EAT burden in T2DM patients, Jonker and colleagues reported an increased pericardial fat volume within the same population [43, 79]. Additionally, pioglitazone has been shown to reduce MMP‐9 levels, which may contribute to plaque stabilization by attenuating extracellular matrix degradation and fibrous cap vulnerability [80].

#### 3.2.4 SGLT2 Inhibitors

Selective sodium–glucose cotransporter 2 inhibitors (SGLT2is) confer well‐established cardiovascular benefits, including reductions in major adverse cardiovascular events and mortality in high‐risk populations [81]. Empagliflozin has shown favorable effects on EAT, including reduced EAT volume and modulation of epicardial preadipocyte differentiation and secretome profile [44, 82]. Furthermore, SGLT2i treatment blunts the systemic TNF‐α and CRP response, increases serum adiponectin, and elicits desirable changes in circulating lipid levels, which may collectively promote adaptive remodeling of EAT, as reported for empagliflozin and dapagliflozin [45]. SGLT2 is primarily expressed in preadipocytes within EAT, where dapagliflozin improves the differentiation of epicardial adipocytes, boosts insulin sensitivity, and upregulates GLUT4 [83]. This agent represses pro‐inflammatory cytokine expression and inhibits paracrine‐mediated atherosclerosis. These effects attenuate lipid overflow, lipotoxic stress, and oxidative injury from EAT toward the underlying coronary arteries [84, 85]. Dapagliflozin was associated with a favorable trend toward improved myocardial perfusion reserve in a pilot trial, although the between‐group difference did not reach statistical significance [86].

Notably, dapagliflozin reduced MRI‐derived EAT thickness by approximately 13% over 12 months, independently of changes in body weight and BMI [47].

Even scarce clinical evidence on less commonly used SGLT2i suggests beneficial outcomes. In an MRI study by Bouchi and colleagues, luseogliflozin administration (2.5–5.0 mg/day) yielded a reduction in EAT volume at 12 weeks. The extent of decrements correlated with a drop in systemic CRP levels [49]. Small‐scale studies on canagliflozin (100 mg/day) and ipragliflozin (50 mg/day) have similarly demonstrated marked reductions in EAT thickness and volume [50, 51]. Further studies on less‐studied SGLT2i are noteworthy, as preliminary data reveal agent‐specific differences.

#### 3.2.5 GLP‐1 Receptor Agonists

EAT expresses glucose‐dependent insulinotropic polypeptide receptor, glucagon‐like peptide‐1 receptor (GLP‐1R), and glucagon receptor, particularly within inflammatory CLSs. Endogenous GLP‐1 is an incretin that improves glucose‐dependent insulin secretion and epicardial adipocyte insulin sensitivity; however, its circulating levels are elevated in CAD patients, positively correlating with increased EAT thickness [87, 88]. GLP‐1R activation enhances adipogenesis, brown adipocyte differentiation, and FFA β‐oxidation, supporting a favorable balance of FA storage within EAT and limiting spillover to adjacent coronary arteries [89]. Furthermore, GLP‐1R agonists exert pleiotropic protective actions, including the promotion of M2 macrophage polarization, increased expression of adiponectin, and improved eNOS activity [90, 91]. Both semaglutide (1 mg once weekly) and dulaglutide (1.5 mg once weekly) have been reported to suppress EAT inflammation in preclinical and clinical studies. In particular, patients with higher levels of EAT inflammation were more responsive to these agonists [92, 93]. In a 6‐month study of obese T2DM patients with poor glycemic control, semaglutide enhanced EAT endocrine activity by increasing the release of the antithrombotic protein gelsolin and modulating neutrophil function [93]. Moreover, semaglutide reduced FABP4 levels, suggesting anti‐inflammatory and anti‐atherosclerotic advantages. With ongoing treatment, the profile shifts toward long‐term anti‐inflammatory status [94]. In the STOP randomized trial, semaglutide reduced EAT volume and increased EAT density after 12 months; however, the mechanisms underlying its cardiovascular benefits remain uncertain [52]. Interestingly, liraglutide appears to exert heterogeneous responses on EAT in individuals with T2DM. Several studies have reported EAT thinning of up to 36%, while others reported no statistically significant changes in EAT thickness [54, 55]. According to a recent meta‐analysis, GLP‐1R agonists demonstrated more pronounced decreases in EAT compared with SGLT2 inhibitors or statins. Younger patients with higher BMI experienced considerable EAT reductions [29]. The pharmacological impacts of other GLP‐1R agonists, such as lixisenatide and albiglutide, on EAT function have not been fully evaluated.

#### 3.2.6 DPP‐4 Inhibitors

Dipeptidyl peptidase‐4 (DPP‐4), highly expressed in EAT, is a multifunctional glycoprotein that mainly regulates glucose metabolism by enzymatically degrading GLP‐1 and GIP [95].

DPP‐4 inhibition modulates EAT inflammation by reducing macrophage infiltration and pro‐inflammatory cytokine production, which may indirectly improve coronary endothelial function and plaque stability [96].

Sitagliptin is the most extensively studied DPP‐4 inhibitor regarding its effects on EAT content and function. These effects appear to be independent of systemic weight loss or glycemic control [97].

To ascertain whether there was a measurable clinical benefit, Lima‐Martínez and colleagues conducted an interventional study for T2DM patients insufficiently treated with metformin monotherapy. Fixed‐dose combination of sitagliptin/metformin (50/1000 mg twice daily) resulted in an approximately 15% reduction in EAT [60].

In agreement with these findings, integrating DPP‐4 inhibitors with lifestyle interventions, such as a heart‐healthy diet and regular physical activity, demonstrates potentially synergistic efficacy [34].

However, safety considerations, particularly regarding heart failure risk observed with some DPP‐4 inhibitors, warrant further clinical investigation.

#### 3.2.7 Anti‐Inflammatory Therapies

In a prospective observational study involving 134 patients with moderate‐to‐severe psoriasis, 1 year of systemic biologic pharmacotherapy targeting IL‐12/17/23 or TNF‐α, significantly reduced CCTA‐derived FAI compared with matched controls, independent of lipid levels or conventional risk factors. The most prominent effect was observed in the RCA, highlighting the efficacy of systemic immune pathway blockade in dampening coronary inflammation [61].

In a pilot randomized controlled trial, the 15‐week administration of orticumab, a monoclonal antibody targeting oxidized LDL, lowered pericoronary FAI in patients with psoriasis without necessarily demonstrating change in total EAT quantity. Preliminary results suggest oxidized LDL inhibition may mitigate residual inflammatory cardiovascular risk [62].

Colchicine was associated with a significant reduction in EAT volume on serial CCT in the EKSTROM trial among patients with stable CAD, with no significant changes in density.

In the same trial, colchicine was also associated with reduced progression of overall coronary plaque burden and dense calcified plaque, although the prespecified primary endpoint of low‐attenuation plaque was not significantly changed [63].

## 4 Conclusion

EAT may influence distinct CAD phenotypes through complex interconnected immunometabolic mechanisms. This review addresses the interface between systemic metabolism and local coronary pathology in contemporary cardiovascular practice. Although several pharmacologic classes appear to modify EAT, most available data originate from small‐scale or surrogate endpoint studies. Large‐scale, outcome‐driven RCTs are therefore necessary to determine whether modification of EAT translates into meaningful reductions in the risk of different CAD phenotypes or is merely epiphenomenal. In this context, addressing the possibility of reverse causality is crucial; indeed, it must be considered that the characteristics of EAT could be the result, rather than the cause, of underlying CAD.

## Author Contributions

Seyed Amirhossein Dormiani Tabatabaei wrote the first draft of the manuscript and contributed to the investigation and conceptualization. Mehrnazsadat Dormiani Tabatabaei contributed to the manuscript revision and editing. The authors critically revised the manuscript. They read and approved the final manuscript.

## Funding

The authors have nothing to report.

## Ethics Statement

This manuscript is a review article and does not involve any original research with human participants or animals. Therefore, no ethical approval was required for this work.

## Consent

This review does not involve human participants. Therefore, no informed consent was required for this work.

## Conflicts of Interest

The authors declare that they have no competing interests associated with this manuscript.

## Data Availability

The data that support the findings of this study are available from the corresponding author upon reasonable request.
